# GAPS AND OPPORTUNITIES TO IMPROVE ACCESS TO HEALTHCARE FOR OLDER RURAL VETERANS

**DOI:** 10.1093/geroni/igz038.1425

**Published:** 2019-11-08

**Authors:** Bret Hicken, Aaron T Seaman, Lauren Moo

**Affiliations:** 1 Veterans Rural Health Resource Center-SLC, Salt Lake City, Utah, United States; 2 Iowa City VA Healthcare System, Iowa City, Iowa, United States; 3 Geriatric Research Education & Clinical Center, Bedford, Massachusetts, United States

## Abstract

Almost 25% of US military veterans live in rural areas and over 70% of these are age 55 years or older. The physical, cognitive, and functional declines common to aging coupled with accumulated physical and psychological traumas often incurred through military service make caring for older Veterans an especially difficult challenge. Furthermore, the geographic distances associated with rural living pose a significant barrier to timely access to care for aging Veterans and caregivers living in remote communities. Significant gaps remain in VA’s understanding of the needs of this population, which is essential for developing adequate models of care. This symposium highlights various aspects of the rural older Veteran experience and will suggest opportunities for improving access to care for this population. The first presentation estimates the differential impact of rural status on mortality rates among older Veterans using mortality data from the General Social Survey (Wilmoth et al). A second study is an analysis of urban/rural differences in PTSD symptoms as function of multiple co-factors and accounting for geographic location (Kurth et al). Seaman et al present qualitative data regarding the experience of Veterans with head and neck cancer to understand how rurality impacts cancer survival (Seaman et al). The final presentation will highlight a model to address mental health problems in older Veterans in primary care (Geri-PACT [Kube et al]) and will specifically highlight how this VA program may be adapted to address such needs the needs of older Veterans in rural areas.

